# The role of microRNAs in acrylamide toxicity

**DOI:** 10.3389/fnut.2024.1344159

**Published:** 2024-02-22

**Authors:** Mina Homayoonfal, Danial Molavizadeh, Sara Sadeghi, Reza Sharafati Chaleshtori

**Affiliations:** ^1^Research Center for Biochemistry and Nutrition in Metabolic Diseases, Kashan University of Medical Sciences, Kashan, Iran; ^2^School of Medicine, Kashan University of Medical Sciences, Kashan, Iran; ^3^Research Committee, Kashan University of Medical Sciences, Kashan, Iran

**Keywords:** acrylamide, microRNAs, neurotoxicity, cancer, *in vitro*, *in vivo*

## Abstract

The chemical compound known as Acrylamide (AA) is employed in different industries worldwide and is also found in thermal-processed food. AA has been acting as a reproductive toxicant, carcinogen, and neurotoxic in various animals, which may promote several toxic impacts in animal and human species. Up to now, various studies have focused on the harmful mechanisms and intervention actions of AA. However, the underlying mechanisms that AA and its toxic effects can exert have remained uncertain. MicroRNAs (miRNAs) are a class of short, non-coding RNAs that are able to act as epigenetic regulators. These molecules can regulate a wide range of cellular and molecular processes. In this regard, it has been shown that different chemical agents can dysregulate miRNAs. To determine the possible AA targets along with mechanisms of its toxicity, it is helpful to study the alteration in the profiles of miRNA regulation following AA intake. The current research aimed to evaluate the miRNAs’ mediatory roles upon the AA’s toxic potentials. This review study discussed the AA, which is made within the food matrix, the way it is consumed, and the potential impacts of AA on miRNAs and its association with different cancer types and degenerative diseases. The findings of this review paper indicated that AA might be capable of altering miRNA signatures in different tissues and exerting its carcinogen effects.

## 1 Introduction

Acrylamide (AA) is a small organic molecule in a white crystalline solid form at ambient temperature and pressure. This compound has light sensitivity and is polymerized under ultraviolet irradiation to form polyacrylamide (1). The International Agency for Research on Cancer (IARC) categorized AA as a “probable carcinogen” compound in 1994, and it has been clarified in 2002 that food materials rich in carbohydrates and containing protein are susceptible to forming multiple levels of AA during high-temperature food processes (more than 120°C) including frying and baking (2). Prior to this date, the prevailing belief was that the risk associated with AA primarily stemmed from workplace exposure. This perception was rooted in the extensive use of AA as an industrial substance, notably in the manufacturing of polyacrylamide for applications such as soil conditioning, wastewater treatment, cosmetics, and in the paper and textile industries, dating back to the 1950s. Additionally, AA was identified in cigarette smoke, with each cigarette containing approximately 1.1–2.3 micrograms of AA (3).

During high-heat operations like baking and frying, rich-carbohydrate food compounds are subject to the Maillard reaction, the AA creation’s predominant mechanism (4–6). The level of AA in food materials owning abundant amounts of starch and other carbohydrates crucially depends on thermal processing procedures, and it has been evidenced that within the bounds of a specific temperature span, the part of AA positively correlated with the time and temperature of the thermal process (7, 8). However, boiled or non-heat-treated food apparently lacks AA (9). Moreover, AA can also be observed in consumer goods like food packaging materials, caulking, and adhesives (6, 10).

Recently, it has become evident how AA is formed and exerts mutagenic and carcinogen effects. The AA’s tolerated daily intake (TDI) has been reported to be 2.6 and 40 g/kg/day for carcinogenic and neurotoxic effects, respectively (3, 11). Studies on animals and epidemiology have shown that AA, which is classified as a category 2A carcinogen and is thus likely carcinogenic in humans, may have genotoxic, carcinogenic, neurotoxic, and reproductive effects (12, 13).

Based on the statement of the Commission Regulation (EU) 2017/2158 report appointed benchmark levels and mitigation measures in reducing the content of AA within food products, the accepted average AA value within various processed food materials such as cereals, coffee or potato products must be placed within the 40–4,000 μg/kg range (14).

Therefore, understanding AA’s toxic mechanisms has recently received much attention worldwide. MicroRNAs (miRNAs) are known as endogenous, single-strand, short (20–25 nucleotides non-coding RNAs that can be affected by a variety of substances, including heavy metals, furans, heavy benzenes, and heterocyclic amines (15, 16). It was predicted that about 4% of the human genome codes more than 400 miRNAs, which are assessed to modulate over 30% of all human genes. Despite clarifying the particular function of a few numbers of miRNAs, convincing evidence has demonstrated that miRNAs have notable regulatory functions in a lot of biological pathways (17). Generally, miRNAs induce its degradation or translational suppression by interacting with target mRNAs’ 3’-UTR (3’ untranslated region) part. Furthermore, aberrant regulation of miRNAs may interfere with pivotal cellular functions such as appropriate oxidative balance. For instance, investigations demonstrated that miRNA regulation is significantly affected by PhIP [2-amino-1-methyl-6-phenylimidazo (4,5-b) pyridine] in rats (15). Additionally, exposure to PhIP may result in triggering chronic inflammation by altering the modulation of glutathione S-transferases (18), cyclooxygenases (19), and nuclear factor kappa-B (NF-κB) (20). Chen et al. (21) indicated miR-133a negatively regulated the apoptosis and can be nominated as a potential indicator of benzene toxicity, while Dong et al. (22) demonstrated miR-34a upregulated through a time-dependent way along with positively modulated apoptosis within rat with exposure to furan.

However, the underlying mechanisms of AA toxicity have remained obscure. Furthermore, the principal biomarkers reflecting AA toxicity have not been identified to evaluate efficient interventions. Hence, it is crucial to recognize the pathway through which AA can exert its toxicity effect. This review aimed to investigate the role of miRNAs in AA toxicity both *in vitro* and *in vivo*. Detecting the possible processes and mechanisms related to the miRNAs’ function in AA-induced cytotoxicity may lead to the inhibition and AA toxicity therapy along with the bioactive food materials’ production versus AA.

## 2 Human exposure to acrylamide

There are various sources that humans may be exposed to AA, such as dermal, inhalation, and oral exposure. In addition to thermal-processed food, smoke from cigarettes is one non-dietary trigger of AA that people who smoke, as well as those who do not, may come into contact with. In smokers, cigarette smoke may represent a more important source of acrylamide than diet (23, 24). Moreover, many individuals may be exposed to AA in the occupation via inhalation and dermal absorption, owing to a wide range of AA applications in different industries. Thus, overall exposure to AA depends on the level of contact with other sources such as diet, drinking water, smoking, and workplace. (25). Food items that have been baked, fried, or deep-fried, such as bread, cake, French fries, and potato chips, are thought to have the most AA (Table 1).

**TABLE 1:** The value of acrylamide (μg/kg) in some heat-processed food materials (6, 26).

Food products	Minimum value (μ g/kg)	Maximum value (μ g/kg)
Fried noodles	3	581
Fried rice	< 3	67
Rice crackers, grilled or fried	17	500
Canned black olives	123	1,925
Prune juice	53	267
Fried vegetables	34	34
Nuts	28	339
Coffee (roasted)	45	975
Coffee substitute	116	5,399
Coffee extract/powder	195	4,948
Fish and seafood products, crumbed or battered	< 2	39
Meat/poultry products, crumbed or battered	< 10	64
Chocolate products	< 2	826
Corn crisps	120	220
Bread	10	130
Breakfast cereals	11	1,057
Bread (toast)	25	1,430

Based on the report of Joint Expert Committee on Food Additives, prominent food materials providing AA absorption in the majority of nations are potato chips (16–30%), potato crisps (6–46%), bread (10–30%), coffee (13–39%) as well as pastry and sweet biscuits (10–20%). Due to monomer residues, packaging materials for polyacrylamide might potentially expose people indirectly to AA (6, 27). Even though non-food sources are regarded as one of the main ways of AA intake, it is assumed that diet is the most significant reason for AA exposure in non-smoking people, where about 30% of calorie intake is supplied by food ingredients containing AA (28, 29).

A systematic review and meta-analysis showed that AA content in popcorn varied between 1,017.7–106 μg/kg (30). Also, microwaved popcorn had the lowest levels of AA compared to other preparation methods. Based on the meta-regression, the type of popcorn was an adequate criterion for the AA concentration, and sweet popcorn had higher AA values. The total AA concentration within popcorn has been evaluated as 459.6 ± 220.3 μg/kg (30). Žilić et al. (31) reported a value of AA in thermally processed corn-based food highly consumed within the market globally that AA concentration in corn flakes, corn/tortilla chips, and popcorn was in the range of 5–6,360 μg/kg < limit of quantification (LOQ) to 2,220 μg/kg, and < LOQ to 1,186 μg/kg, respectively.

The outcomes of an investigation on tracking AA levels in different agri-food materials, announced independently by various researchers and projects like the UK Food Standard Agency (32) and CONTAM (33), demonstrated that levels of AA in some agri-food products exceeded form the reference value lately set by Commission Regulation (EU) 2017/215 (29). Accordingly, the mean values of the upper bound/reference levels of AA in μg/kg in some food products are biscuits (637/350), crackers (637/400), breakfast cereals (744/300), coffee substitutes (1,897/500), dried coffee (523/400), French fries (550/500), potato crisps and snacks (2,214/750), and processed cereal-based baby foods (76/40). Thus, it is estimated that the average content of daily exposure to AA via food sources in various is almost 0.4–1.9 μg/kg body weight. However, multiple investigations (including Romanis) have reported that these values can vary between 1.4 and 3.4 μg/kg in different European countries (29).

## 3 Metabolism of acrylamide

**FIGURE 1:**
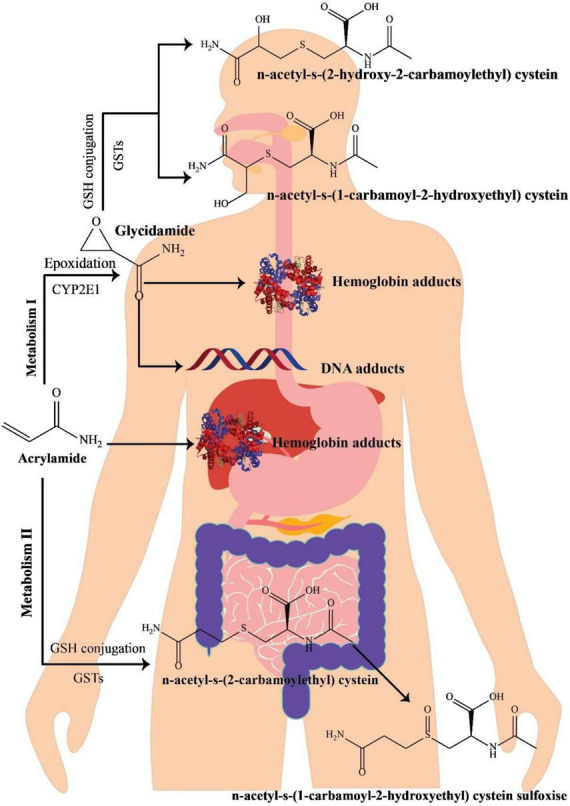
A schematic illustration of acrylamide metabolism in the human body, adapted from Zhao et al. (41).

Various investigations on the metabolism of AA in rats have indicated that following oral administration through circulation, the gastrointestinal system wholly and quickly absorbs AA, which is then distributed to the peripheral tissues. It seems that the metabolic pathway of AA in humans is approximately comparable with that in rodents (Figure 1) (34). Preliminary research on healthy cases has revealed that AA may simply pass the blood-placenta barriers within an *in vitro* model of human placenta and also the blood-breast milk barriers within the *in vivo* model of breastfeeding moms, suggesting that AA owns the capability of reaching anywhere in the human body (35). After absorption, AA is metabolized via two principal pathways. Thus, it is capable of being converted to N-acetyl-S-(3-amino-3-oxopropyl) cysteine via glutathione-S-transferase (GST) or might be changed into glycidamide under the mechanism mediated through a cytochrome P450 enzyme complex (CYP450). In contrast to the original AA compound, the subsequent metabolite has a higher propensity to engage with DNA and proteins (36, 37). This heightened interaction is linked to its genotoxic properties, leading to carcinogenic effects (38). Approximately 6% of a consumed AA dosage undergoes conversion into the glycidamide epoxide (3, 39). Glycidamide demonstrated its capacity to hinder progesterone production by inducing reactive oxygen species (ROS) and triggering apoptosis in R2C Rat Leydig Cells (40).

The process of metabolizing and detoxifying AA involves its conjugation with GSH, resulting in the formation of GSH adducts. These adducts undergo swift conversion into their corresponding mercapturic acid metabolites, namely, N-acetyl-S-(3-amino-3-oxopropyl)-cysteine (AAMA), N-acetyl-S-(1-carbamoyl-2-hydroxyethyl)-cysteine (ISO-GAMA), and N-acetyl-S-(2-carbamoyl-2-hydroxyethyl)-cysteine (GAMA). Ultimately, these metabolites are expelled from the human body through urine (34, 39). Since mercapturic acid metabolites are the main metabolic products of AA and glycidamide, their levels in urinary excretion are regarded as AA exposure’s biomarker (42). AA and glycidamid may also help hemoglobin’s amino acids and DNA create adducts. Accordingly, such adducts are AA exposure’s distinctive hallmarks (43). In 2002, mice treated with fried meal ingredients significantly increased the value of hemoglobin adducts, highlighting the relevance of AA as a dietary contaminant (2). Furthermore, due to the existence of AA-hemoglobin conjugate in newborn blood, AA can pass the placental barrier (44).

The onset of AA toxicity initiates when there is a disruption in the equilibrium between biological oxidants and antioxidants, giving rise to oxidative stress. This stress arises from an excess of oxidants, leading to the deterioration of cellular macromolecules and, ultimately, culminating in cell death through apoptosis (3). A prior investigation indicated that exposure to AA and glycidamide resulted in reduced cell viability and heightened levels of oxidative stress and apoptosis in Leydig and Sertoli cells. *In vitro* findings strongly suggest that oxidative stress likely plays a pivotal role in the apoptosis induced by AA and glycidamide in these cells (45). Studies have documented that hydroxylamine, potentially derived from acrylohydroxamic acid treated with amidase, undergoes autoxidation through the Cu(II)/Cu(I) redox cycle, leading to the generation of H2O2. This observation implies that acrylamide-related carcinogenesis is significantly influenced by oxidative DNA damage induced by ROS. Furthermore, the administration of amidase-treated acrylohydroxamic acid demonstrated a dose-dependent elevation in the formation of 8-oxo-7,8-dihydro-2’-deoxyguanosine in calf thymus DNA. This serves as an indicator of dose-dependent oxidative DNA damage (38).

Additionally, previous investigations demonstrated that the AA level in most tested cases (human breast milk) never exceeded 0.5 μg/L (46). A study evaluated the status of AA circulation in two breastfeeding women after consuming food materials rich in AA. Results disclosed that the AA levels varied between 3.17–18.8 μg/L based on the elapsed time after eating, suggesting that the diet of nursing women substantially impacts the AA content within breastmilk. In addition, the length of time since the mother last fed affects the amount of AA in breast milk at the time of collection (47).

## 4 Acrylamide affecting miRNA profiles

### 4.1 An introduction to miRNAs biogenesis

**FIGURE 2:**
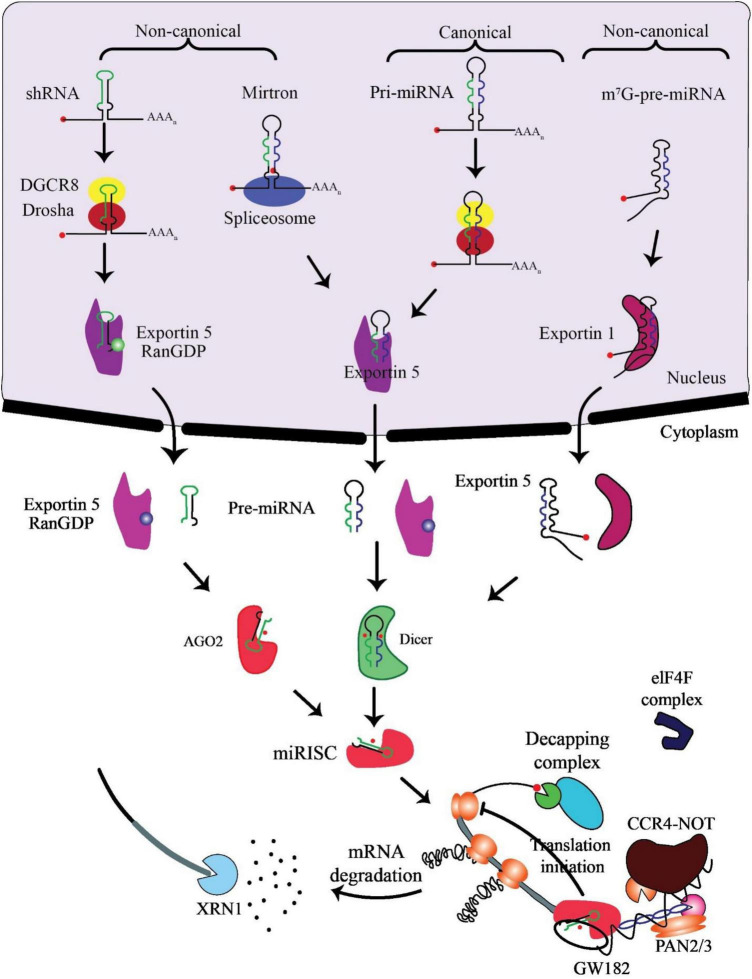
Biogenesis and mechanism of miRNAs. The production of the pri-miRNA transcript marks the beginning of canonical miRNA biogenesis. The pri-miRNA is broken down through the microprocessor complex made up of DiGeorge Syndrome Critical Region 8 (DGCR8) and Drosha to create the precursor-miRNA (pre-miRNA). Thus, the pre-miRNA has been processed to create a mature miRNA duplex within the cytoplasm in a way that is reliant on Exportin5 and RanGTP. The mature miRNA duplex is then loaded onto a member of the Argonaute (AGO) family of proteins to create a miRNA-induced silencing complex (miRISC). Small hairpin RNA (shRNA) has been first cut via the microprocessor complex in the non-canonical pathways and then transported to the cytoplasm by Exportin5/RanGTP. They undergo more processing via cleavage that relies on AGO2 but not Dicer. Dicer is required for the 7-methylguanine capped (m7G)-pre-miRNA and the mirtrons’ cytoplasmic maturation, although their nucleocytoplasmic shuttling differs. While Exportin1 exports m7G-pre-miRNA, Exportin5/RanGTP exports mirtrons. A functioning miRISC complex has been the end outcome of every possible path. Most frequently, target mRNAs are bound by miRISC to prevent translation; probably by interacting with the eIF4F complex, this is achieved. The poly (A)-deadenylases CCR4-NOT and PAN2/3 are then recruited through GW182 family proteins that are coupled to Argonaute. The decapping complex might remove the target mRNA’s m7G cap since deadenylation, initiated by PAN2/3 and completed by the CCR4-NOT complex, has taken place. The exoribonuclease XRN1 might then cause decapped mRNA’s 5’–3’ degradation, adapted from O’Brien et al. (55).

miRNA biogenesis involves co- or post-transcriptional processing of RNA polymerase II/III transcripts for synthesis initiation (48). Intergenic miRNAs, independent of host genes, are controlled by their promoters, while around half of protein-coding genes rely on introns for intragenic miRNA processing (49). MiRNAs, translated as clusters, form families when including related seed regions, with synthesis pathways categorized as canonical and non-canonical (Figure 2).

In the canonical pathway, Drosha and DGCR8 collaborate to transform pri-miRNAs into pre-miRNAs, exported to the cytoplasm via Exportin5/RanGTP and cleaved by Dicer. The resulting mature miRNA duplex, loaded onto AGO proteins, has strand bias influenced by factors like 5’ uracil and thermodynamic stability (50). AGO2 mediates cleavage of passenger strands, impacting the strand bias. Non-canonical pathways, including Dicer-independent and Drosha/DGCR8-independent processes, introduce further complexity, generating pre-miRNAs such as mirtrons and m7G-capped pre-miRNAs (51). These pathways diversify miRNA biogenesis beyond the canonical process (52).

Several studies have displayed that miRNAs considerably impact oncogenesis and cancer prevention via the degradation of the target mRNAs. Additionally, abnormal regulation of miRNAs is related to various kinds of cancer (53, 54). Furthermore, several studies have disclosed that AA exerts its toxic effects via altering miRNA regulation (Table 2).

**TABLE 2:** Summary of toxicological effects of acrylamide (AA) both *in vitro* and *in vivo*.

Study	Model	Survey	Intervention	Duration	Result
(56)	Female 7-8-weeks-old Sprague-Dawley (SD) rats	Mitochondrial dysfunction and intrinsic apoptosis	High doses: Oral administration (35 mg/kg)	21 days	– ↑ miR-27a-5p – Knockdown of *Btf3* – Phosphorylation of ATM and p53
			Oral administration (10 mg/kg)	120 days	
(57)	Male Swiss mice	Epididymal toxicity	Intraperitoneal injection of acrylamide (25 mg/kg/day)	5 days	– ↑ miR-20a-5p, ↑ miR-30a-5p and ↑ miR-30b-5p in the caput epididymis
(58)	Liver cell (BRL-3A)	Cytotoxicity	100 mM	24 h	– ↓ miR-193b-5p
(13)	Female 7-weeks-old SD rats	Alters the miRNA profiles in various tissues	Oral administration (35 mg/kg)	17 days	– ↑ miR-143-3p in brain, kidney and hepar – ↑ miR-122-5p in hepar – ↑ miR124-3p in brain – ↑ miR-33a – 5p in kidney – ↑ miR-21a-3p hepar, bladder, lung, kidney, stomach and uterus – ↑ miR-21a – 5p in hepar and lung – ↑ miR-27a-5p especially in bladder and hepar
(54)	Liver cell (HepG2 cells)	Cytotoxicity	≤ 100 mmol/L	4 h	– ↑ miR-21 expression

### 4.2 The interplay between acrylamide and various miRNAs

#### 4.2.1 miRNA-193b-5p

Yang et al. (58) exhibited that AA was able to decrease the miRNA(miR)-193b-5p expression in BRL-3A cells, causing induced cell cycle at the G1/S phase along with cell proliferation through overexpression of the cyclin-dependent kinase regulator Cyclin D1 along with cyclin-dependent kinase inhibitor p21 downregulation. Further investigation disclosed that miR-193b-5p modulated proliferation and cell cycle by targeting FoxO3 at 3’-UTR, which promoted the regulation of p21 and Cylin D1. Therefore, MiR-193b-5p reduces AA’s cytotoxic effects in BRL-3A cells.

It has been found that the miR-193b-5p has been downregulated within interleukin-1β-stimulated osteoarthritis while miR-193b-5p overexpression directly decreased the HDAC7 modulation (59). By targeting Cyclin D1 and cell cycle progression through binding to the PLK1 mRNA’s 3’UTR, down-regulation of miR-193b-5p also played a crucial part in malignant phenotypes of lung cancer (60, 61). Uncertainty surrounds miR-193b-5p’s critical function in cells exposed to AA.

Yang et al. (58) exhibited that AA was able to decrease the miR-193b-5p expression within BRL-3A cells, causing induced cell cycle at the G1/S phase as well as cell proliferation through overexpression of the cyclin-dependent kinase regulator Cyclin D1 as well as downregulation of cyclin-dependent kinase inhibitor p21. Further investigation revealed that miR-193b-5p modulated proliferation and cell cycle by targeting FoxO3 at 3’-UTR, which promoted the regulation of p21 and Cylin D1. The Foxo transcription factor family includes FoxO3, a common transcription factor engaged in many biological procedures like cell division and proliferation. It was reported that FoxO3 expression by numerous miRNAs. miR-193b-5p upregulation prevented the FoxO3 expression, and treating BRL-3A cells with miR-193b-5p mimics successfully stopped vector-FoxO3-induced cell growth and reversed cell cycle arrest at the G1 phase. Furthermore, miR-193b-5p mimics via targeting the siFoxO3 can affect the cell cycle and proliferation, while miR-193b-5p inhibitor exerted adverse effects. Thus, miR-193b-5p is necessary for modulating the cytotoxic effects of AA within BRL-3A cells by inhibiting the FoxO3 regulation from inducing cell cycle and proliferation by Cyclin D1 upregulation and reducing the p21 expression.

Numerous investigations have indicated that AA induces a reduction in the proliferation of various cells *in vitro*. One study noted a significant inhibition at a concentration of 4.0 mmol/L for 12 h (90.2 ± 4.5%) or 2.0 mmol/L for 24 h (87.7 ± 4.4%). Furthermore, the cell count notably dropped to less than 30% following exposure to concentrations exceeding 6.0 mmol/L of acrylamide for 24 h. The IC50 value of acrylamide in RAW 264.7 cells was determined to be 4.9 mmol/L after 24 h (62). A preceding investigation demonstrated that the treatment of Caco-2 cells with AA (ranging from 0.2 to 50 mM) resulted in a decline in cell viability that was both time- and dose-dependent over 24 to 72 h. At the highest AA concentration (50 mM), cytotoxicity exceeded 84.0%–94.4% in the MTT (3-(4,5-dimethylthiazol-2-yl)-2,5-diphenyltetrazolium bromide) assay and 78.4%–82.2% in the PrestoBlue assay after exposure for 24–72 h (63). Another investigation indicated that AA led to a decline in the proliferation of undifferentiated C17.2 and SH-SY5Y cells. In SH-SY5Y cells, the attenuation of the differentiation process was observed at AA concentrations starting from 10 fM, sustaining cell proliferation. Neurite outgrowth was reduced at concentrations from 10 p.m. Moreover, AA significantly decreased the number of neurons starting at 1 μM and altered the ratio between different phenotypes in differentiating C17.2 cell cultures. Additionally, their findings revealed that the toxicity of AA is directly correlated with the duration of exposure (64). In an experiment, it was illustrated that as the concentration of AA increased from 1 to 6 mg/ml, the viability of HeLa cells decreased in a dose-dependent manner, reaching from 76 to 30% within a 24-h period (65).

#### 4.2.2 miRNA-21

A previous study reported that AA-induced HepG2 cell proliferation was inhibited by miR-21 inhibitor. The miR-21 level moderately altered slightly in combination with AA and miR-21 inhibitors. All results confirmed that it is a vital component in the proliferation of HepG2 cells promoted by AA. AA increased the HepG2 cell proliferation via modulating expression of PTEN and Akt through upregulation of miR-p21. Cyclin D1 and EGFR were two proliferation-associated proteins whose overexpression was caused by AA’s upregulation of p-Akt. Further research has shown that LY294002, an inhibitor of Akt/PI3K signaling, reduced the expression of Akt’s downstream targets while inhibiting Akt’s phosphorylation (54).

Moreover, transfection of the miR-21 inhibitor into HepG2 cell lines overexpressed PTEN and downregulated p-Akt and its downstream genes. Therefore, AA reduced the PTEN expression, upregulated p-Akt and induced HepG2 cell proliferation. Additionally, AA-induced antiapoptotic effects through overexpression of Bcl-2 and downregulation of Bax. Incubation of HepG2 cells with curcumin reduced AA-stimulated cell proliferation via elevated expression of miR-21 and apoptosis induction.

#### 4.2.3 miRNA-27a-5p

Typically, two 21-nucleotide miRNAs with completely comparable base sequences, miR-27a-5p and miR-27a-3p, can be produced from the miR-27a precursor (pre-miR-27a). The miR-21a-30 expression significantly increased in AA-treated rats in six tissues, specifically liver tissue (13). According to reports, miR-27a-5p and miR-21-3p act together directly to influence the NF-kB signaling axis (66). Additionally, some investigations indicated that AA may cause neurotoxicity by activating NF-kB signaling (67). A research study evaluated the effects of AA on the expression of miRNAs by treating rats with a high AA dosage (35 mg/kg/day, 17 days) or a low AA dosage (3.5 mg/kg/day, 68 days) (13). Their studies’ results demonstrated that miR-27a-5p expression within bladder tissue has been substantially elevated within both high-dosage AA-treated rats (about 100 times) along with low-dosage AA-treated rats (about 5-9 times) compared to the control groups with no AA treatment. Hence, both high and low dose of AA treatment can promote the bladder damage in rats.

Adani et al. (68) disclosed that consumption of high levels of AA was correlated with increased risks of ovarian and endometrial cancer. It was also declared that miR-27a-5p was remarkably overexpressed in AA-treated rats’ uterine and ovarian tissues (13). Furthermore, the current investigation revealed for the very first time that miR-27a-5p expression surprisingly increased in the stomach about 7-8 times. Formerly, only one investigation illustrated that high and low doses of AA supplementation were associated with alteration in the porcine stomach’s neurotoxic symptoms (69). Although the stomach has been an essential site for food digestion in people, it has attracted insufficient interest as a target tissue for AA toxicity. Therefore, they asserted that more attention should be paid to stomach damage caused by the consumption of AA, which indicates the importance of emphasizing more on the problems of AA in food (13).

**FIGURE 3:**
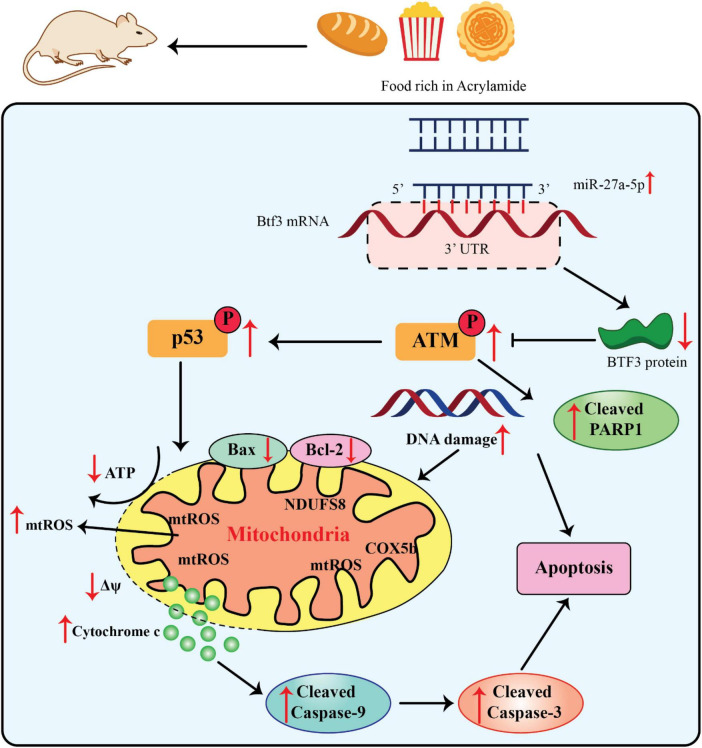
Acrylamide promoted cell apoptosis via the miR-27a-5p-Btf3-ATM-p53 signaling pathway, adapted from Zhang et al. (76).

The miR-27a’s 5’arm of the stem-loop sequence precursor forms miR-27a-5p. Current investigations have concentrated on the modulation of hypoxia-induced liver and kidney injuries and different kinds of cancer (70, 71). The considerable dysfunction of mitochondria caused diminished Δψ and ATP generation as well as mitochondria-related apoptosis via upregulation of the cleaved caspase-9 and -3 and Bax/Bcl-2 ratio (Figure 3).

Since miR-27a-5p is derived from the 5’ arm of the pre-miR-27a stem-loop sequence, recent research on this miRNA has concentrated on how it controls kidney and liver injury in hypoxia as well as various malignancies (70, 71). The miR-27a-5p expression was significantly elevated within the liver as well as serum of AA-treated rats, along with the development of neurotoxic phenotypes. Moreover, the miR-27a-5p expression was directly related to the intensity of AA-promoted apoptosis. Thus, this finding significantly contributed to the estimate of AA exposure (13, 56). Btf3 was recognized as an indication of upregulation in different kinds of cancers i.e., lung cancer and breast cancer as well as neurodegenerative diseases. It has also been applied as an efficient hallmark for identifying the progress stage of colorectal cancer and Alzheimer’s diseases (72–75). The upregulation of Btf3 attenuated the impairment of the mitochondrial activities, namely, AA-promoted oxidative stress, suggesting that it can be a substantial therapeutic target in different interventions and an essential modulator of mitochondria practice in AA toxicity (56).

### 4.3 Effects of acrylamide on small non-protein-coding regulatory RNA profiles

Small non-protein-coding regulatory RNA (SncRNAs) have different functions, including gene modulation by RNA interference, RNA modification, or spliceosomal involvement. Therefore, their regulation may change during various disorders. miRNAs are the most well-known sncRNA indicators involved in different health problems, including aging, cancer, and neurodegenerative diseases. Other types of sncRNAs are valuable therapeutic biomarkers related to neurodegenerative disorders. Accordingly, various sncRNAs can be introduced as potential biomarkers for neurodegenerative diseases and may help in therapeutic diagnosis in the clinical context while disclosing the mechanisms supporting the disease progress (77).

Although it has been proved that paternal exposure to different environmental stressor factors prompted apparent alterations in the sncRNAs profile of sperm with unique post-fertilization subsequences, the underlying associated mechanisms have remained unrevealed. Researchers evaluated the acute sensitivity effects of AA as a reproductive toxicant on the sncRNA landscape of sperm. Moreover, they tracked the distinctive accumulation of AA-reactive sncRNAs to be concurrent with the transition of sperm to the proximal (caput) section of the epididymis, in which AA exposure changes the levels of different transcription factors involved in the AA-responsive sncRNAs expression (57). The outcomes of the current investigation demonstrated that exposure of the sperm to AA during testicular development (AA-S) lost to regulate the adequate of miR-30b-5p, miR-30a-5p, miR-20a-5p within the sperm. At the same time, all of them have been overexpressed within the sperms exposed to AA during the epididymal transition (AA-E). Remarkably, these miRNAs were downregulated within the somatic epithelium encompassing such cells inside the caput epididymis. Yet, the caput epithelial cells’ reaction to AA exposure was dynamic; 3 days after the final AA injection, the regulation of AA-sensitive miRNAs changed in a mutually beneficial manner and showed a substantial rise, suggesting a counterbalance reaction to the discontinuation of the AA treatment.

Furthermore, 6 days after AA injection, the modulation of miRNAs promisingly returned to the control values, suggesting the capability of the epididymis to retrieve succeeding the termination of AA treatment (57). They reported that both biochemical detoxifications of glycidamide by-products of AA could induce changes in the epididymis soma proteome (57). Such reactions indicated compelling counterparts with other hippocampus and central nervous system cells, where AA promoted considerable alterations within the proteome (78, 79). Although the primary mediators of such responses are undisclosed, it was assumed that transcription factors might be the feasible central modulators. In-silico studies suggested that at least seven transcription factors were upregulated in AA-exposed caput epididymal epithelial cells, namely, NR3C1, RBFOX2, STAG1, NCOR2, RELA, MBD3, and CTCF. Hence, these transcription factors may be modulated by miRNA genes, among which the AA-sensitive ones changed in the AA-E spermatozoa were provided (57).

The acute exposure to AA impacted the profiles of sncRNA in the spermatozoa of mice and assigned significant mechanistic association considering both the origin and subsequent of a mutated sncRNA sperm profile. While it seemed that a crucial part of this response was the distinctive reactivity of the epididymal cells to acute paternal damages, it was revealed that this tissue’s dynamic features are responsible for rapidly reacting following ending stress. This type of dynamic response has been reflected in investigations related to human spermatozoa, suggesting the profile of sncRNAs may be changed within a week following paternal exposure to food intake (13, 57).

## 5 Conclusion

In summary, this review reported AA’s influence on miRNA dysregulation in both animal and cell line models. These changes might impact substantial targets in different signaling pathways, resulting in disease progression. Substantial development has been achieved in realizing the effects of AA in miRNA regulation and cancer progress. However, there is no agreement on a stable miRNA profile for AA-promoted cancer and degenerative diseases, mainly because most studies investigate specific miRNAs rather than the entire genome. On the contrary, the role of miRNAs in AA-promoted non-cancer disorders has attracted weak attention, a crucial issue that should be considered in future studies. A particular miRNA profile might be beneficial as an indicator for evaluating the exposure, diagnosis, and therapeutic approaches. Moreover, this review study foregrounded the common alterations in miRNA regulation among rodents and cell lines, which might apply as guidance for choosing miRNAs for future investigations.

## Author contributions

MH: Investigation, Validation, Writing–review and editing. DM: Investigation, Methodology, Writing–original draft. SS: Investigation, Methodology, Writing–original draft. RC: Investigation, Methodology, Supervision, Validation, Writing–original draft, Writing–review and editing.
